# A Rare Presentation of Nocardia pericarditis Leading to Cardiac Tamponade in an Immunocompetent Patient

**DOI:** 10.7759/cureus.4140

**Published:** 2019-02-26

**Authors:** Erika L Faircloth, Patrick Troy

**Affiliations:** 1 Internal Medicine, University of Connecticut, Farmington, USA; 2 Pulmonology, Hartford Hospital, Hartford, USA

**Keywords:** cardiac tamponade, alcohol abuse, pericardial effusion, immunocompetent patient, nocardia farcinica

## Abstract

*Nocardia* can cause isolated disease in many parts of the body including the brain, skin, and lungs. It is also capable of causing disseminated disease. In almost all cases, *Nocardia* infections occur in immunocompromised hosts with depressed cell-mediated functions. We present a case of disseminated *Nocardia farcinica* leading to pericardial effusion and tamponade in an immunocompetent host with the only risk factor being heavy alcohol intake. Treatment relies on an accurate diagnosis. This case presentation highlights the importance of considering *Nocardia* infections in an alcoholic patient with a worsening clinical picture.

## Introduction

*Nocardia* species are gram-positive, filamentous, environmental saprophytic rod-shaped bacteria [1-3]. Nocardiosis is rare and can present as acute bronchopneumonia, cutaneous pustules, or disseminated disease [2-3]. Nocardia pericarditis is very uncommon, with only a few documented cases due to *Nocardia farcinica* (*N. farcinica*) [4-5]. The majority of *N. farcinica* infections occur in immunocompromised patients [6-8]. We describe a case of *N. farcinica* pericarditis, leading to pericardial tamponade in an immunocompetent host with alcohol use disorder as the only predisposing risk factor. Because diagnosis requires specially ordered microbiologic mediums and delay in diagnosis and treatment can result in significant morbidity and mortality, consideration of *N. farcinica* early in the presentation is crucial.

## Case presentation

**Figure 1:**
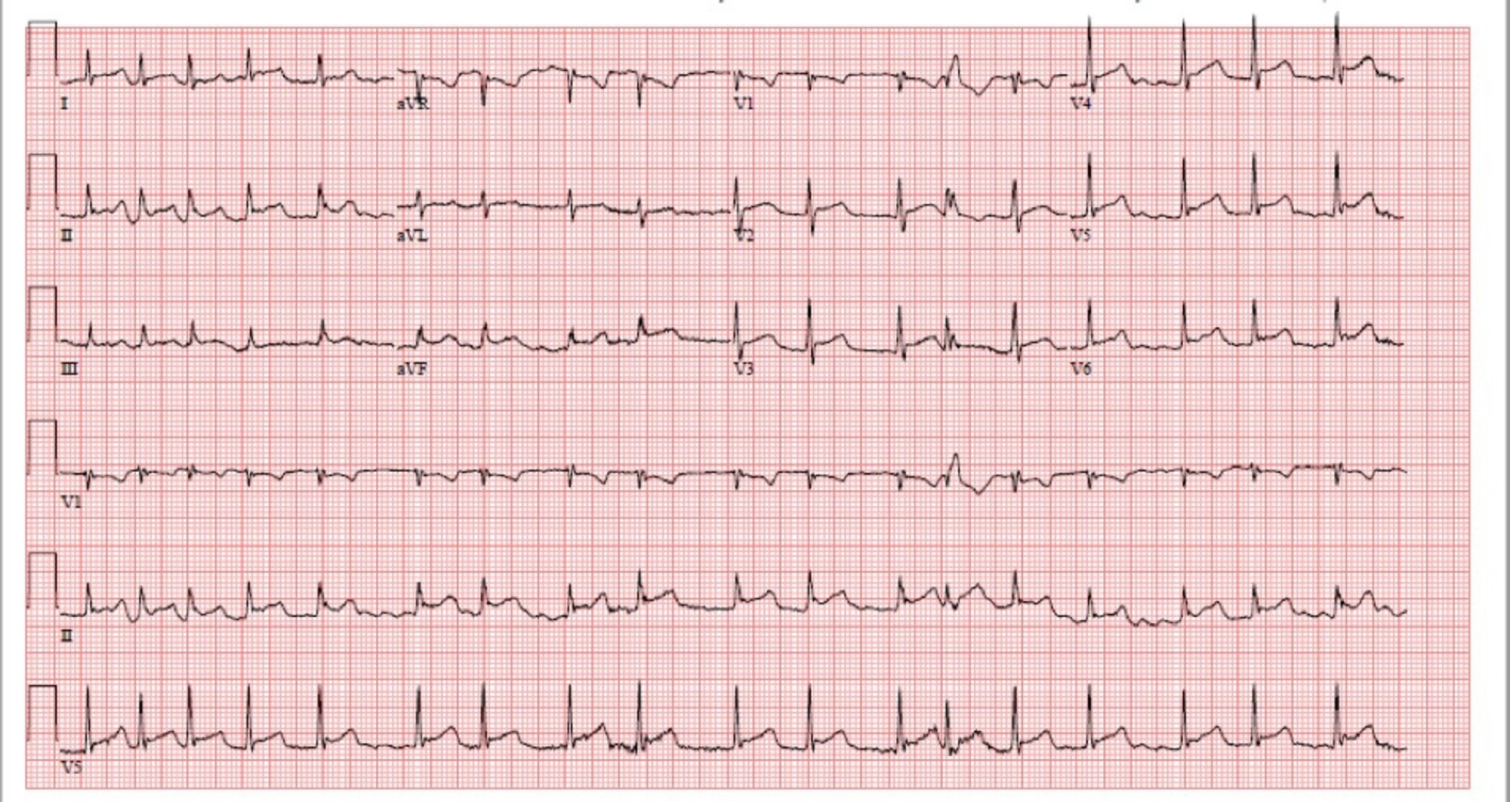
EKG showing atrial fibrillation and diffuse ST elevations EKG: electrocardiogram

A 60-year-old man with a history of heavy ethanol abuse presented with three weeks of worsening shortness of breath associated with positional chest pressure improved by sitting forward. He denied other upper respiratory symptoms including nasal congestion, sore throat, or cough. An electrocardiogram (EKG) showed new-onset atrial fibrillation and diffuse ST segment elevations (Figure 1).

**Figure 2:**
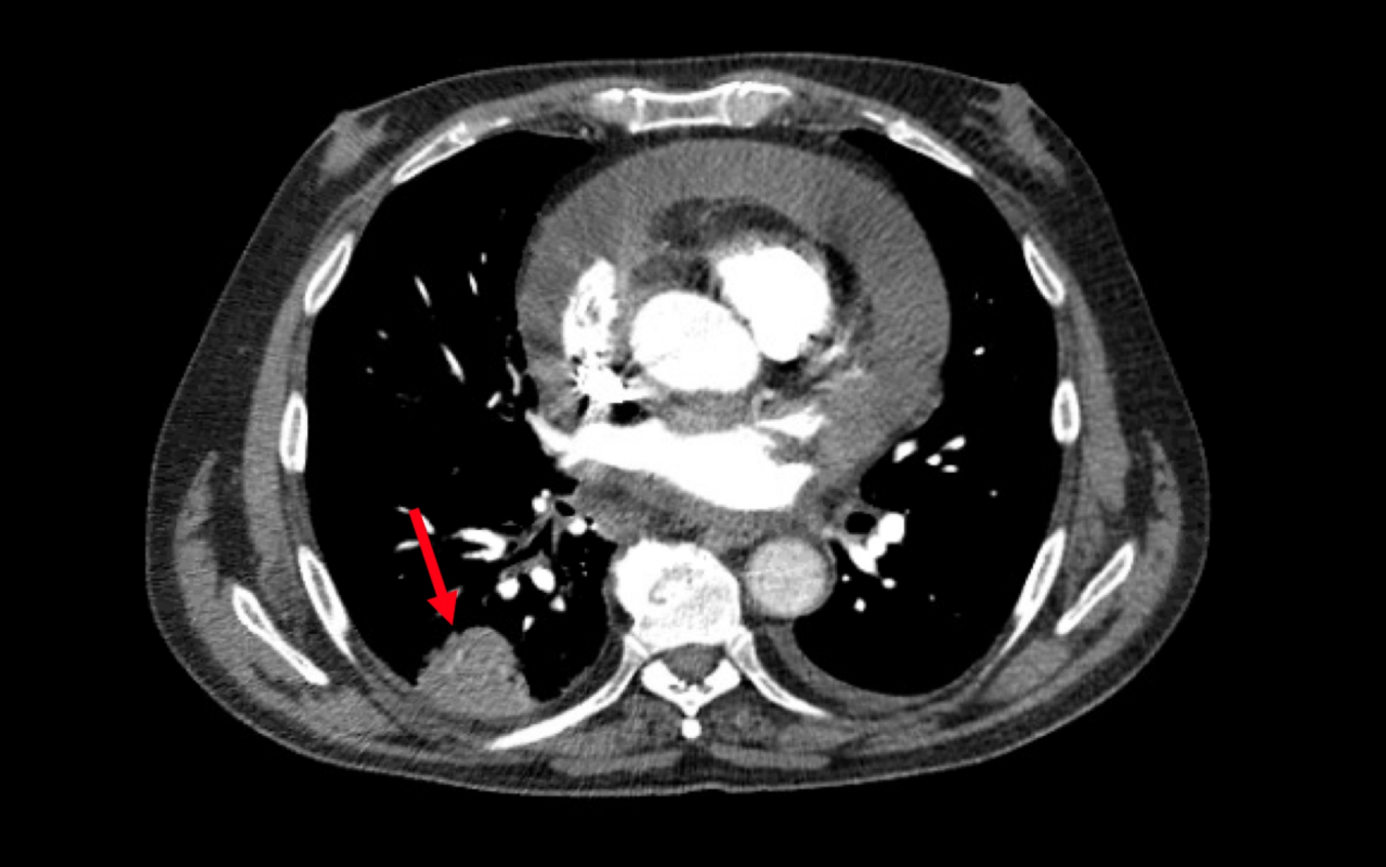
Computed tomography of the chest with the arrow pointing towards a right basilar pulmonary nodule

Subsequently, a transthoracic echocardiogram was done revealing a large, greater than 2 cm, pericardial effusion with greater than 30% variation of mitral inflow velocity with impairment of the right ventricular filling consistent with tamponade physiology. The patient underwent a pericardial window which yielded 300 mL of serous fluid with evidence of epicardial and pericardial inflammation. Pericardial fluid studies were significant for inflammation without an infectious or malignant source at that time. Other studies including human immunodeficiency virus (HIV), antineutrophil cytoplasmic antibodies (ANCA), hepatitis panel, *Ehrlichia chaffeensis* titers, and Lyme titers were all negative. Computed tomography (CT) angiography of the chest ruled out pulmonary embolism but revealed a right lower lobe pulmonary nodule. For the nodule, he underwent a CT-guided lung biopsy demonstrating organizing pneumonia (Figure 2).

Repeat EKG was performed for worsening respiratory distress and demonstrated a moderate pericardial effusion and constrictive pericarditis with severe right ventricular dysfunction. The patient decompensated requiring intubation, Swan-Ganz catheter placement, and vasopressor and inotropic support. At this time, pericardial fluid studies, bronchoalveolar lavage, and respiratory cultures were done earlier started to grow *N. farcinica*, prompting consultation of the infectious disease team and initiation of antibiotics including imipenem/cilastatin, linezolid, and sulfamethoxazole/trimethoprim for disseminated nocardiosis. In this case, the only predisposing factor for disseminated nocardiosis was his chronic alcohol abuse.

## Discussion

*Nocardia *can hematogenously spread to infect many organ systems and can be very difficult to diagnose due to slow growth requiring selective mediums [2,9]. Lung-limited infection occurs in approximately 39% of cases, central nervous system-limited infection in 9% of cases, cutaneous-limited infection in 8%, and disseminated infection occurs in 32% of cases [2]. Very rarely, *Nocardia* can lead to purulent pericarditis requiring surgical drainage [9]. There are several subspecies of *Nocardia* known to cause disease in humans; around 80% of respiratory infections and disseminated infections are caused by *N. asteroides* [5,8,10]. However, *N. farcinica* has a higher propensity for dissemination due to its higher virulence and proneness to antibiotic resistance [7,10].

There are approximately 500 to 1,000 newly diagnosed significant infections due to *Nocardia* species per year in the United States and around two-thirds are in immunocompromised hosts [8,11]. In a review of 52 cases of *N. farcinica* infections, 85% of patients had a predisposing condition for acquiring the infection [6-7]. Cell-mediated impairments predispose patients for *Nocardia* infections such as organ transplants, human immunodeficiency virus, immune suppression, and systemic lupus erythematosus [2-3,9,12-13]. Alcoholism is not one of the frequently cited predisposing conditions; however, alcohol is known to be an immunosuppressant, especially with respiratory infections [14].

We present a rare case of *N. farcinica* leading to pericardial effusion and subsequent tamponade in an otherwise immunocompetent host other than heavy alcohol use. This underscores the importance of identifying alcohol use disorder as a strong risk factor for *Nocardia* infections and screening for it in patients with a worsening clinical picture.

## Conclusions

Early identification of *Nocardia* is essential to provide appropriate treatment. However, because special media is required for identifying *Nocardia*, a high clinical suspicion for the diagnosis is required. *Nocardia* should be considered in patients with worsening clinical status even in immunocompetent patients if they have risk factors such as alcoholism.
